# Effects of a motor control exercise program on lumbopelvic pain recurrences and intensity in pregnant women with a history of lumbopelvic pain: a study protocol for a randomized controlled feasibility trial

**DOI:** 10.1186/s40814-022-01024-0

**Published:** 2022-03-21

**Authors:** Catherine Daneau, Andrée-Anne Marchand, André Bussières, Julie O’Shaughnessy, Stephanie-May Ruchat, Martin Descarreaux

**Affiliations:** 1 Department of Anatomy, Université du Québec à Trois-Rivières, Trois-Rivières, Québec, Canada; 2 Department of Chiropractics, Université du Québec à Trois-Rivières, Trois-Rivières, Québec, Canada; 3 School of Physical and Occupational Therapy, McGill University, Montreal, Canada; 4 Department of Human Kinetics, Université du Québec à Trois-Rivières, Trois-Rivières, Québec, Canada

**Keywords:** Motor control exercise, Pregnancy, Lumbopelvic pain, Prevention, Treatment

## Abstract

**Background:**

About 50% of women experience lumbopelvic pain (LBPP) during their pregnancy. LBPP has negative repercussions on sleep, social and sexual life, physical and work capacity, and psychological health and contributes to physical inactivity. The benefits of LBPP prevention or treatment in pregnant women through specific exercises should therefore be further investigated. This study protocol has been designed to establish the feasibility of implementing motor control exercise program with pregnant women presenting with a history of LBPP.

**Methods/design:**

Forty pregnant women with a history of LBPP will be recruited and randomly allocated to a control (20 participants) or intervention (20 participants) group. The control group will receive standard prenatal care, including basic information on what to do when suffering from LBPP. The intervention group will participate in three 40-min exercise sessions per week from < 20 weeks until 34–36 weeks of gestation: one supervised group session via the Zoom platform (once a month, this session will take place at the *Université du Québec à Trois-Rivières*) and two unsupervised sessions at home. A motor control exercise program will be developed to target strengthening of the lumbo-pelvic-hip core muscles and improve spinal and pelvic stabilization. Participants of this group will also receive standard prenatal care. Women of the control group will receive after 6 weeks postpartum an exercise program designed to reduce LBPP they may have developed during pregnancy and that may persist after delivery. Primary outcomes will be participants’ recruitment, retention and adherence rates, safety, and acceptability of the intervention. Secondary outcomes will include LBPP incidence, frequency, and intensity, as well as self-reported functional disability, physical activity levels, fear avoidance behavior, anxiety, and depression.

**Discussion:**

This study will inform the feasibility of conducting a full-scale randomized controlled study to test the effectiveness of a motor control exercise program on the prevention and treatment of LBPP in women with a history of LBPP. Adequate prevention and treatment of pregnant women with a history of LBPP should help limit the recurrences of LBPP or the aggravation of its intensity during pregnancy.

**Trial registration:**

US National Institutes of Health Clinical Trials registry NCT04253717 April 27, 2021.

**Supplementary Information:**

The online version contains supplementary material available at 10.1186/s40814-022-01024-0.

## Background

Approximately 50% of women experience low back pain (LBP) or pelvic girdle pain (PGP) during pregnancy, and 25% will still experience pain one year after delivery [1]. A 10-year follow-up study reported that 1 in 10 women who had PGP during pregnancy still has severe consequences up to 11 years after delivery [1–5]. LBP is defined as pain or discomfort located between the 12th rib and the gluteal fold, whereas PGP has been defined as “pain experienced between the posterior iliac crest and the gluteal fold” [6]. When pregnant women have both types of pain (i.e., LBP and PGP), the pain is commonly referred to as lumbopelvic pain (LBPP). Indeed, there seems to be a consensus (despite variation in definition) that the term LBPP is used when no distinction is made between LBP and PGP [5].

Several hypotheses have been suggested to explain the development of LBPP during pregnancy. Hormonal, anatomical, biomechanical, and postural changes likely interact in the development of pregnancy-related LBPP [6–8]. As the fetus grows, the women’s center of gravity moves towards the abdomen. This results in an increased lumbar lordosis, posterior tilt of the sacrum, and consequent backward extension of the head relative to the back to compensate for the increased lumbar lordosis and weight [9–11]. Moreover, the increased lumbar lordosis is believed to not only change the load distribution between the different structures of the spine, but also to increase the facet joint load sharing contribution, which would contribute to LBPP [12]. Accordingly, a study published in 2003 reported that pregnant women with LBPP suffered more from postural constraints, such as a forward shifting of the trunk center of mass, than pregnant women without LBPP [13]. Inefficient neuromuscular control is believed to contribute to the etiology of LBPP in pregnant women [6]. One study investigated LBPP intensity and disability in pregnant women with and without LBPP before pregnancy [14] and found a significant correlation between current pain intensity and lumbar muscles activity level during trunk flexion. The study also reported that paraspinal muscles activity during bending measured in the first trimester of pregnancy was significantly correlated with pain intensity measured in the third trimester [14].

One important risk factor for developing LBPP during pregnancy is having a history of LBPP [15]. Furthermore, early LBP in pregnancy appears to be a predictor for long-term PGP [2]. Interestingly, a comprehensive review reported that the mean prevalence of postpartum LBPP was higher in studies of pregnant women with a history of LBP (42.7%) compared to when they were excluded from studies (24.3%) [5]. It is therefore of paramount importance to gain knowledge on these women because the literature shows that pregnant women with a history of LBPP have a threefold increased risk to develop LBPP during their pregnancy compared to pregnant women without a history of LBPP [5].

Despite its high prevalence, LBPP during pregnancy is often considered a common and almost natural phenomenon which often leads to inadequate clinical prevention and treatment of the condition [16]. LBPP has negative repercussions on sleep, social and sexual life, physical and work capacity, as well as on psychological health [17, 18]. Importantly, LBPP also contributes to physical inactivity during pregnancy [19], which has been associated to a higher incidence of maternal and neonatal complications [20–23]. Despite the frequent occurrence of LBPP during pregnancy and the significant impact it can have on the women’s quality of life, there are currently no specific guidelines available to inform clinical prevention and treatment of this condition. Not surprisingly, several studies indicate that about 25 to 30% of women suffering from LBPP self-manage or seek a wide range of interventions delivered by various care providers to prevent and treat their symptoms [24, 25]. However, the evidence regarding these interventions is limited due to restricted availability of high-quality studies and very low evidence strength [26, 27]. It is therefore essential to identify effective clinical strategies to prevent and treat pain and disability, while considering the safety of both the mother and growing fetus.

The recently published *2019 Canadian guideline for physical activity throughout pregnancy* [20] clearly highlights that, not only is exercise safe for both the mother and the growing fetus, but that it also improves key pregnancy outcomes such as decreased risk of gestational hypertension and diabetes [22], excessive gestational weight gain [23] and depression [28]. The guideline summarizes the best available evidence and provides consensus-based recommendations on physical activity during pregnancy. It suggests that women without any medical restriction should accumulate at least 150 min of a variety of moderate-intensity physical activity each week to achieve clinically meaningful reduction in pregnancy complications, such as gestational diabetes mellitus, gestational hypertensive disorders, and prenatal depression [21]. Nonetheless, the guideline does not provide any specific recommendation for the prevention and treatment of LBPP during pregnancy, mainly due to the lack of evidence regarding the effect of prenatal exercise on the occurrence of pregnancy-related LBPP and the poor quality of evidence (i.e., “very low”) and heterogeneity of the data regarding its effect on LBPP intensity [22]. Considering that a history of LBPP is one of the strongest predictors of LBPP during pregnancy [15] and that it is a debilitating condition associated with significant personal, social, and economic burden [17, 18, 29], exercise may offer a safe and cost-effective self-management strategy option to decrease the recurrences and symptom intensity of LBPP for expecting mothers with a history of LBPP. However, more robust research is needed to identify suitable or effective exercise modalities to optimize self-management of LBPP in these women.

The primary objective of the study described in this protocol is to assess the feasibility of implementing a motor control exercise program with pregnant women presenting a history of LBPP in order to reduce LBPP recurrences or limit its intensity. The secondary objective is to assess the potential effectiveness of the program. The first hypothesis is that the motor control exercise program will show high recruitment, adherence rates as well as low attrition among pregnant women with a history of LBPP in the intervention group. The second hypothesis is that the proposed motor control exercise program will reduce LBPP recurrences or limit its intensity.

## Methods/design

### Design

This study is a parallel randomized controlled feasibility trial involving two groups of pregnant women with either a history of LBPP or current LBPP. Participants are randomized to either standard prenatal care or standard prenatal care combined with a motor control exercise program (allocation ratio 1:1). It has been designed to assess the feasibility and the potential effectiveness of a motor control exercise program to reduce LBPP recurrences or limit its intensity in pregnant women with a history of LBPP. This study protocol complies with the guidelines of the SPIRIT checklist [30]. The proposed study protocol has been approved by the institutional review boards of the *Université du Québec à Trois-Rivières* (CER-19-259-07.20) and *Centre Intégré Universitaire de Santé et de Services Sociaux de la Mauricie-et-du-Centre-du-Québec* (CIUSSS-MCQ, local health services) (CÉRM-2019-004-01). The study will take place at the *Université du Québec à Trois-Rivières* and has been registered at Clinicaltrials.gov (NCT04253717). Before any intervention be initiated, participants will provide written informed consent in accordance with the certification delivered by the institutional review boards.

### Recruitment

Recruitment of participants will be conducted at local medical clinics as well as in the local community via social media including Facebook. Medical teams will talk to pregnant women about the study and, if they are interested in participating, a research team member will meet with them and provide an overview of the study purpose and content. Those willing to participate in the study will be met at the university laboratory for the pre-intervention visit. At this visit, informed written consent will be obtained, and baseline demographics and physical measures will be collected. Participants will also complete questionnaires assessing functional disability, physical activity levels, fear avoidance behavior, anxiety, and depression. At the end of the pre-intervention visit, a member of the research team will open a sealed opaque envelope in front of the participant to reveal the participant’s random allocation to one of the two groups (intervention or control group).

### Eligibility criteria

Pregnant women aged 18–40 years old, carrying one fetus and being ≤ 20 weeks pregnant (based on the ultrasound performed in the first trimester of pregnancy) and presenting with a history of LBPP (currently in pain or not) will be eligible to participate in the study. Women undergoing their first LBPP episode (of at least 2 weeks duration) will also be included. The inclusion criteria are based on the potential full-scale trial objective to reduce LBPP recurrences or limit its intensity in pregnant women with a history of LBPP. To confirm the presence and intensity of LBPP and women’s eligibility for the trial, a medical history and recommended clinical tests [6, 31] will be conducted at the pre-intervention visit (baseline) by a member of the research team. Women presenting any of the following conditions will be excluded from the study: inflammatory rheumatic disease, infectious disease, neuromuscular disease, vascular disease, connective tissue disease, severe disabling pain, and neurologic signs and symptoms. Women presenting contraindications to exercise (according to the 2019 Canadian guideline for physical activity throughout pregnancy [21]) will also be excluded from the study. Women unable to understand or speak French as well as those unwilling to be randomized will also be excluded.

### Sample size

Pregnant women will be recruited and randomly allocated to a control (20 participants) or intervention (20 participants) group. A sample size of 40 pregnant women was chosen based on a previous study we conducted and in which we were able to recruit 40 pregnant women in twelve months [32] and considering that over 4600 women gave birth at the local health services in 2016–2017 (including 1800 at the *CIUSSS-MCQ*) [33]. Furthermore, one of the study goals is to obtain an estimate of variance for the primary outcomes of a full-scale study. A minimally clinical important difference between groups has already been established and it is suggested that 10 to 20 participants per group is sufficient to inform feasibility and to plan for a larger study [34]. Between group differences in LBPP and disability score after the intervention will be used to estimate adequate sample size for future research planning.

### Randomization

At the end of the pre-intervention visit, participants will be randomly allocated to one of the two groups. The randomization sequence generation will be performed by an independent research assistant using a computer random number generator while the allocation sequence concealment will be performed using sequentially numbered, opaque and sealed envelopes. To ensure good balance of factors known to affect the natural history of pregnancy-related LBPP in our small sample, two minimization criteria will be considered: baseline pain intensity and baseline physical activity levels. Participants will not be blinded to intervention allocation, but the content of the exercise sessions will be shared only to those randomized to the intervention group to prevent cross-contamination between groups. The kinesiologist who will prescribe and supervise the exercise sessions will not take part in the pre- and post-intervention evaluations. The member who is running the pre- and post-intervention evaluations and who is managing the database, as well as the person who is supervising the exercise sessions, are not blinded. Members of the research team involved in statistical analyses will be blinded to group allocation.

### Intervention

#### Control group

Pregnant women in the control group will receive standard prenatal care, including basic information on what to do when suffering from LBPP, which is provided in the practical guide *From Tiny Tot to Toddler* *[*35*]. *This guide, published by the *Institut National de Santé Publique du Québec*, is offered at no cost to all pregnant women in the Province of Quebec [35]*.* After 6 weeks postpartum, women will receive an exercise program designed to reduce LBPP they may have developed during pregnancy and that may persist after delivery.

#### Intervention group

Pregnant women in the intervention group will participate in three 40-min exercise sessions per week from ≤ 20 weeks of gestation until 34–36 weeks of gestation: one supervised group session via the Zoom platform (once a month, this session will take place at the *Université du Québec à Trois-Rivières*) and two unsupervised sessions at home.

All sessions will include a 5-min warm-up period followed by specific exercises of moderate-intensity aimed at strengthening muscles of the lumbo-pelvic-hip core complex in order to improve stabilization and alignment of the spine and pelvis (transversus abdominis, internal obliques, multifidus, pelvic floor, thigh, and hip muscles) [36, 37]. The sessions will end with gentle stretching. No specific equipment will be needed to complete the home exercise component of the program. The intervention will be adapted to the stage of pregnancy and related discomfort to ensure a safe, individualized, and yet motivating training experience for each participant. Women will receive a weekly text message with an individually tailored home-exercise program to be completed before the next group session. The text message will contain a link giving access not only to the weekly exercise program, but also to a web-based electronic diary to help participants document their participation in the home exercise sessions (compliance and adverse events), incidence (a painful episode), frequency, and intensity of daily LBPP, as well as any treatment received to prevent and treat LBPP. Participants of this group will also access the same standard prenatal care recommendations as the control group, which includes basic information about what to do when suffering from LBPP, which is provided in the practical guide“ *From Tiny Tot to Toddler”* [35]*.*

### Data collection

#### Primary outcome measures

##### Feasibility components: recruitment, retention, adherence rate, safety of the intervention, and acceptability

The feasibility of recruitment, evaluated for both groups at the pre-intervention visit, will be defined as the ability to recruit eligible women throughout a 12-month period. The feasibility of obtaining a successful retention rate and consequently low attrition will be assessed using the completion of pre- and post-intervention questionnaires (see below). Adherence to the protocol will be defined as attendance to the supervised group exercise sessions and completion of the unsupervised home exercise sessions. The retention and adherence rates will be evaluated for the intervention group at the post-intervention visit. Safety of the intervention will be determined based on the number of adverse events, which will be defined as symptom flare-ups that will prevent a woman from taking part in subsequent exercise sessions or injuries requiring medical attention. In addition to the kinesiologist taking note of any undesirable effects during supervised exercise group sessions, women from the intervention group will be asked to report, via weekly text messages and their web-based diary, any reaction or flare-up that is not consistent with their usual pain presentation as a result of either home exercise or supervised group exercise sessions. Information concerning adverse events will be collected for the intervention group on a weekly basis throughout the study. Acceptability of the intervention, assessed for the intervention group at the post-intervention visit, will be determined based on how the intervention will be perceived by pregnant women (including satisfaction, practicality and accessibility assessed using 5-point Likert scales).

#### Secondary outcome measures

The potential effectiveness of the intervention will be assessed using the following clinical outcomes and assessment tools. All secondary outcome measures (i.e., LBPP incidence, frequency and intensity, functional disability and physical activity levels) will be obtained during the pre-intervention (≤ 20 weeks of gestation) and post-intervention (34–36 weeks of gestation) visit at the university laboratory. Fear avoidance behaviors the level of anxiety, and depression will also be assessed pre- and post-intervention, as they are considered potential prognostic factors (and confounding variables) of LBPP [38].

##### LBPP incidence, frequency, and intensity

Lumbar and pelvic pain presence and intensity will be evaluated using recommended clinical tests [6, 31] and a 100-mm visual analog scale (VAS) [39]. The construct validity of the visual analog scale has been shown to be significantly and positively correlated with other self-reported measures of pain intensity (i.e., 101-point numerical rating scale, 11-point box scale, 6-point behavioral rating scale, 4-point verbal rating scale, and 5-point verbal rating scale) [40, 41]. Furthermore, participants will receive weekly text messages to collect information about the incidence (first episode during pregnancy), frequency (number of days), and intensity of LBPP. The minimal clinically important difference (MCID) for this outcome will be set according to Hagg et al. (2003) who defined the difference in the score change between patients assessing themselves as “better” and those assessing themselves as “unchanged”. The MCID for this outcome will be 18–19 mm on a 100-mm VAS scale [42].

##### Functional disability

Functional disability will be measured using the Pelvic Girdle Questionnaire (PGQ) [43]. The PGQ is a 25-item questionnaire scored on a Likert-type scale that includes a 20-item activity subscale and a 5-item symptoms subscale. Items 1–20 and 23–25 scores range from 0 (not at all) to 3 (to large to extent) while items 21 and 22 scores range from 0 (none) to 3 (considerable) [43]. The MCID for this outcome will be 11 points [44].

##### Physical activity levels

Physical activity levels will be measured using the Pregnancy Physical Activity Questionnaire (PPAQ) [45]. The PPAQ is a self-administrated 33-question questionnaire which provides a comprehensive assessment of four domains of physical activity including “Sports and Exercises” (*n* = 9), “Household and Caregiving” (*n* = 16), “Transportation” (*n* =3), and “Occupation” (*n* = 5) [45].

##### Fear avoidance behavior

The fear avoidance behavior will be measured using the Tampa Scale of Kinesiophobia (TSK) [46]. The TSK comprises 17 questions evaluating the fear of movement and physical activities resulting from being afraid to get hurt; it uses a Likert-type scale ranging from “strongly disagree” to “strongly agree” [47].

##### Anxiety

Anxiety levels will be measured using the State-Trait Anxiety Inventory (STAI-Y) [38]. The STAI-Y comprises two distinct scales: situational anxiety and anxiety trait. The situational anxiety is composed of 20 sentences assessing the current emotional state of the individual. The individual indicates the emotion intensity on a Likert-type scale ranging from “not at all” to “a lot” [38]. The anxiety trait is composed of 20 sentences assessing habitual emotional state of an individual. The individual indicates the frequency with which she usually feels the symptoms on a Likert-type scale ranging from “almost never” to “almost always” [38].

##### Depression

Depression levels will be measured using the Beck Depression Inventory (BDI) [48]. The BDI is a 21-item questionnaire evaluating different specific behavioral manifestation of depression on a Likert-type scale reflecting the severity of depression symptoms [48].

#### Additional assessments

Participants will answer several questions during the pre-intervention visit to collect the following information: age, gestational age, pre-pregnancy weight, and educational level. Height and weight will be measured at the pre- and post-intervention visits.

The timeline illustrating the various interventions and outcome assessments is presented in Table 1.

**Table 1:** Schedule of laboratory visits, intervention and outcomes measures

	Study period			
Timepoint	Enrolment	Pre-intervention	Throughout the study	Post-intervention
**Enrolment:**				
**Eligibility screen**	X			
**Informed consent**	X			
**Allocation**		X		
**Interventions:**				
***Control group***			X^a^	
***Intervention group***			X^b^	
**Assessments:**				
***Primary outcomes measures***				
***Recruitment rate***		X		
***Retention rate***				X
***Adherence rate***				X^c^
***Safety of the intervention***			X^c^	X^c^
***Acceptability of the intervention***				X
***Secondary outcomes measures***				
***LBPP incidence, frequency and intensity***		X	X	X
***Functional disability***		X		X
***Physical activity levels***		X		X
***Fear avoidance behavior***		X		X
***Anxiety***		X		X
***Depression***		X		X

Pre-intervention visit (≤ 20 weeks of gestation); throughout the study (between pre-intervention and post-intervention visits); post-intervention visit (34–36 weeks of gestation)

^a^Standard prenatal care

^b^Motor control exercise program at UQTR and at home as well as standard prenatal care

^c^Intervention group only

### Statistical analysis

Demographic data will be presented using means and standard deviations. For primary outcome measures (recruitment, retention, adherence rate, safety, and acceptability), means, standard deviations and/or percentage will be presented. For secondary outcomes measures, descriptive statistics will be used to describe within-group changes from baseline visit to post-intervention visit (34-36 weeks of pregnancy). Differences between group in mean change with confidence intervals and effect sizes (Cohen’s *d*) will be reported for each measure.

### Progression criteria

The following criteria must be met in order to consider progression to a main RCT:

- Recruitment criteria: recruitment rate of 40 eligible women over a 12-month period.
- Retention criteria: retention rate of ≥ 80% of recruited women.
- Adherence criteria: adherence rate of ≥ 75% to the supervised and unsupervised exercise sessions.
- Safety criteria: less than 25% of women experiencing acute LBPP flare-ups interfering with daily activities.

## Discussion

In the general population, there is “moderate” quality evidence that exercise has a small positive effect on the intensity of chronic LBP compared to usual care [49]. The effect size is similar to other non-pharmacological approaches recommended in the non-pregnant population for the prevention and treatment of acute or chronic LBP [49]. Compared to other cost-effective non-pharmacological prevention and treatments (e.g., acupuncture, spinal manipulation, interdisciplinary rehabilitation, or cognitive-behavioral therapy), exercise is easily accessible as part of a self-management strategy, requires minimal equipment, and can be performed at home [50]. However, the effect of exercise on LBPP (incidence and intensity) in pregnant women remains unclear. A recent systematic review and meta-analysis assessed the effect of prenatal exercise on maternal LBP, PGP, and LBPP during pregnancy [51]. According to their results, the articles selected for this study had poor quality evidence and had some methodological issues such as small sample size, high dropout rates, lack of compliance with the exercise interventions, and poor reporting on the use of co-interventions [51]. The authors concluded that various types of prenatal physical activity had a large effect on decreasing the severity of LBP, PGP, and LBPP during pregnancy but had no effect on the odds of developing these conditions [51]. Since pregnant women with LBPP seem to have decreased stability of pelvic girdle joints [6], an exercise program with specific stabilizing exercises may bring more benefits for those women [52]. Indeed, a recent study showed that a lumbar stabilization and stretching program was effective in reducing LBPP in pregnant women [53]. Therefore, a motor control exercises program may be ideal since it includes coordination and balance exercises [54].

The study explores a pragmatic approach for the prevention and treatment of LBPP through a motor control exercise program combining supervised (university laboratory) and unsupervised (home) exercise sessions. Considering that the pregnant women included in this study have either a history of LBPP or are currently experiencing LBPP, secondary prevention for this population is obviously relevant. This motor control exercise program will allow pregnant women to progress at their own pace through different levels of exercises. In addition, the use of technology in the follow-up of the women will make the approach flexible and adapted to their daily routines.

This feasibility study aims to provide preliminary data to inform a future full-scale clinical study investigating the effects of a motor control exercise program in pregnant women presenting a history of LBPP. It is essential to understand the impact of such an exercises program on the women's quality of life and behaviors, as well as on maternal and fetal health since many women will experience LBPP during their pregnancy and even after delivery.

### Trial status

The recruitment of pregnant women has begun in April 2021.

## Supplementary Information

**Additional file 1.** SPIRIT 2013 Checklist: Recommended items to address in a clinical trial protocol and related documents.

## Data Availability

Data sharing is not applicable to this article as no datasets were generated or analyzed during the current study.

## Abbreviations

BDI: Beck Depression Inventory
CIUSSS-MCQ: Centre Intégré Universitaire de Santé et de Services Sociaux de la Mauricie-et-du-Centre-du-Québec
LBP: Low back pain
LBPP: Lumbopelvic pain
MCID: Minimal clinically important difference
PGP: Pelvic girdle pain
PGQ: Pelvic Girdle Questionnaire
PPAQ: Pregnancy Physical Activity Questionnaire
STAI-Y: State-Trait Anxiety Inventory
TSK: Tampa Scale of Kinesiophobia
UQTR: *Université du Québec à Trois-Rivières*
